# Preclinical assessment of MAGMAS inhibitor as a potential therapy for pediatric medulloblastoma

**DOI:** 10.1371/journal.pone.0300411

**Published:** 2024-10-22

**Authors:** Zahra Motahari, Javier J. Lepe, Malia R. Bautista, Clay Hoerig, Ashley S. Plant-Fox, Bhaskar Das, Christie D. Fowler, Suresh N. Magge, Daniela A. Bota

**Affiliations:** 1 CHOC Neuroscience Institute, Children’s Hospital of Orange County, Orange, CA, United States of America; 2 Department of Pediatrics, University of Irvine, Irvine, CA, United States of America; 3 Department of Neurology, School of Medicine, University of Irvine, Irvine, CA, United States of America; 4 Department of Neurobiology and Behavior, School of Biological Sciences, University of California, Irvine, CA, United States of America; 5 Department of Pediatric Oncology, Children’s Hospital of Orange County, Orange, CA, United States of America; 6 Department of Pediatrics, University of Minnesota, Minneapolis, MN, United States of America; 7 Department of Pediatric Oncology, Ann and Robert H. Lurie Children’s Hospital of Chicago, Chicago, IL, United States of America; 8 Arnold and Marie Schwartz College of Pharmacy and Health Sciences, Long Island University, Brooklyn, NY, United States of America; 9 Department of Medicine and Pharmacological Sciences, Icahn School of Medicine at Mount Sinai, New York, NY, United States of America; 10 Department of Neurosurgery, Children’s Hospital of Orange County, Orange, CA, United States of America; 11 Department of Neurosurgery, University of Michigan, Ann Arbor, MI, United States of America; Goethe University Hospital Frankfurt, GERMANY

## Abstract

Medulloblastoma is the most common malignant brain tumor in children. It has WNT-driven, SHH-driven/*TP53* mutant, SHH-driven/*TP53* wildtype, and non-WNT/non-SHH subgroups. MAGMAS (Mitochondrial Associated Granulocyte Macrophage colony-stimulating factor Signaling molecules) encodes a mitochondrial import inner membrane translocase subunit and is responsible for the translocation of matrix proteins across the inner membrane. We previously reported that a small molecule MAGMAS inhibitor, BT9, decreases cell proliferation, migration, and oxidative phosphorylation in adult glioblastoma cell lines. The aim of our study was to investigate whether the chemotherapeutic effect of BT9 can be extended to pediatric medulloblastoma. **Methods:** DAOY (SHH driven/tp53 mutant) and D425 (non-SHH group 3) were treated with BT9. For *in vitro* analysis, cell proliferation, death, migration, invasion, and metabolic activity were assessed using MTT assay, TUNEL staining, scratch wound assay, Matrigel invasion chambers, and seahorse assay, respectively. A D425 orthotopic xenograft mouse model was used to evaluate BT9 efficacy in *vivo*. **Results:** BT9 treatment resulted in a significant decrease in cell proliferation (DAOY, 24 hours IC50: 3.6 μM, 48 hours IC50: 2.3 μM, 72 hours IC50: 2.1 μM; D425 24 hours IC50: 3.4 μM, 48 hours IC50: 2.2 μM, 72 hours IC50: 2.1 μM) and a significant increase in cell death (DAOY, 24 hours p = 0.0004, 48 hours p<0.0001; D425, 24 hours p = 0.0001, 48 hours p = 0.02). In DAOY cells, 3 μM BT9 delayed migration and significantly reduced DAOY and D425 cell invasion (p < 0.0001). It also modified mitochondrial respiratory function in both medulloblastoma cell lines. Compared to control, however, BT9 administration did not improve survival in a D425 orthotopic xenograft mouse model. **Conclusions:** Our *in vitro* data showed BT9 antitumor efficacy in DAOY and D425 cell lines, suggesting that BT9 may represent a promising targeted therapeutic in pediatric medulloblastoma. These data, however, need to be further validated in animal models.

## Introduction

Medulloblastoma is the most common pediatric brain malignancy and occurs mainly in children aged between 3 and 9 years [1,2]. Standard therapy for medulloblastoma includes surgical resection, craniospinal irradiation, and a combination of chemotherapeutic agents [3]. These treatments, however, can lead to severe side effects such as growth failure, and cognitive deficits, and subsequent untreatable secondary malignancies [3]. Drug resistance is also very common, which severely limits the effectiveness of current chemotherapy [1]. Therefore, a more effective treatment approach is required to overcome the development of drug resistance and to improve clinical outcomes.

Medulloblastoma represents a heterogenous tumor that can be divided into WNT-driven, SHH-driven/*TP53* mutant, SHH-driven/*TP53* wildtype, and non-WNT/non-SHH groups. The non-WNT/non-SHH class can be further subdivided to group 3 (G3) and group 4 (G4) when it is possible to distinguish the two [4]. Although each group has a different molecular signature, aberrant MYC proto-oncogene (C-, N-, and L-) amplification seems a common theme in SHH, G3, and G4 [5]. N-MYC is highly expressed in SHH, G3, and G4, and C-MYC shows overexpression in the most aggressive G3 and G4 [5]. MYC proteins are considered the master regulators of cellular programs such as proliferation, apoptosis, and cell size. MYCs control metabolism through their regulatory effects on glycolytic genes such as hexokinase and lactate dehydrogenase [6]. In addition, MYC have been reported to regulate mitochondrial biogenesis by inducing genes involved in inner membrane, carboxylic acid metabolism, oxidative phosphorylation, and mtDNA replication [7,8]. The inner membrane proteins upregulated by MYC include TIM8 (translocase of the inner mitochondrial membrane), TIM10, TIM13, TIM17A, TIM22, TIM23, TIM44, and TIM16 or MAGMAS (S1 Fig) [7,9]. As MYC is one of the most “undruggable” targets in cancer biology, alternative approaches targeting downstream pathways might prove useful on the MYC-dependent malignancies such as medulloblastoma [10].

TIM16 or MAGMAS (Mitochondrial Associated Granulocyte Macrophage colony-stimulating factor Signaling molecules) is an ortholog of yeast pam16 (presequence translocase-associated protein import motor) and highly conserved in eukaryotes [11]. MAGMAS encodes the MAGMAS/TIM16 subunit of the mitochondrial inner membrane translocase. Alongside its partners, including TIM17, TIM18, TIM44, Hsp70, and Mge1, MAGMAS forms an essential part of the TIM23 import complex (TIM17, TIM23, and TIM50). Together, these complexes facilitate the ATP-dependent translocation of proteins across the inner mitochondrial membrane into the matrix [12,13].

MAGMAS expression controls the production of Reactive Oxygen Species (ROS) in cells [14]. Overexpression of MAGMAS leads to decreased ROS and increased cellular tolerance to oxidative stress, whereas its downregulation increases cellular ROS levels and increased susceptibility to ROS-mediated apoptosis [14]. Several studies have demonstrated the involvement of MAGMAS in human diseases. A homozygous missense mutation in MAGMAS correlates with severe skeletal dysplasia [15]. The expression of MAGMAS is elevated in human, murine, and rat pituitary adenoma cell lines as well as in samples from patients with prostate and ovarian cancer and glioblastoma [16–20]. Single-cell RNA seq and microarray data further reveal the increased expression of PAM16 in medulloblastoma subtypes (S2 Fig) [21].

BT9 is a small molecule MAGMAS inhibitor, synthetized as a yellowish-white or white crystal that has a melting point of 165–167°C [22]. It binds to TIM14-TIM16 heterodimer interface in the mitochondrial import machinery, resulting in complex dissociation [22]. We previously showed that BT9 decreases cell proliferation, migration, and oxidative phosphorylation in human glioblastoma cell lines and can cross the blood brain barrier [19]. Since glioblastoma and childhood brain tumors differ clinically in their pathophysiology [23], the aim of this study was to investigate whether the therapeutic effect of MAGMAS inhibition can be extended to pediatric medulloblastoma.

## Methods and materials

### Cell culture

The established SHH-activated/tp53 mutant (DAOY) [24], and G3 (D-425) medulloblastoma cell lines were cultured in DMEM/F-12 medium containing 10% fetal bovine serum (FBS) (Cat # 12306C, Sigma), and 1% Penicillin/streptomycin (Cat # 15140–122, Gibco). All cells were tested for mycoplasma infection and maintained at 37°C in a humidified incubator with 5% CO2. The BT9 compound was synthesized in Dr. Das’ laboratory as previously published [22] and dissolved in DMSO and Captisol (Ligand Pharmaceuticals, San Diego) for *in vitro* and *in vivo* studies, respectively.

### Cell viability assay

The cells were seeded at approximately 5x 10^3^/well in a final volume of 200 μl in 96-well microtiter plates and allowed to grow for 24 h before the addition of BT9 compound. 5mg/ml MTT solution (Cat # 50-213-524, Fisher Scientific) was added at 24, 48, and 72 hours after BT9 treatment and cells were incubated at 37°C for 4 hours. The culture medium was then aspirated and DMSO (200 μl/well, Fisher Scientific) was added to dissolve the dark blue formazan crystals. Absorbance was measured at a wavelength of 570 nm. Three independent experiments with 3 replicates per condition were performed. Data are expressed as relative survival compared to DMSO, and IC50 was determined using non-linear regression analysis on effect-log concentration curves.

### Apoptosis assay

DAOY apoptotic cells were detected by Click-iT^TM^ TUNEL Assay in accordance with the manufacturer’s instructions (Invitrogen, Cat# C10247). 2.5 × 10^4^ cells were seeded in a Nunc Lab-Tek chamber slides system (Fisher Scientific, Cat# 177445) and treated with daily BT9 for 24 and 48 hours. Slides were imaged with a Nikon Ti-E microscope and analyzed using Image J. More than 3000 nuclei were counted per field; the experiment was repeated three times.

### Migration assay

Cells (6 x 10^5^) were seeded in a 6-well plate to form a confluent monolayer. A scratch wound was made in the center of the well by scraping the cell layer with a P200 pipette tip. Cells were then washed with PBS, treated with 10%FBS + BT9 compound, and incubated to allow cells to migrate into the space cleared by the tip. Cell migration was assessed by capturing images using an EVOS X10 microscope at 0,4,8, and 12 hours after the scratch.

### Transwell invasion assay

Invasion assays were performed using a 24-well Matrigel Invasion chamber (Corning, USA) containing an 8μm pore size polyethylene terephthalate (PET) membrane treated with Matrigel basement membrane matrix (Cat # 354480). DAOY and D425 (2.5 × 10^4^) cells were pretreated with 3 μM BT9 and plated in the top chamber in serum-free medium. Cells were allowed to invade the lower chamber (containing culture medium with 10% FBS) for 22 hours. Then, the nonmigratory cells were removed from the upper side of the membrane, and the cells on the lower surface of the membrane were fixed and stained with 0.5% crystal violet. The number of migratory cells was determined by counting the cells that had penetrated the membrane using a Nikon Ti-E microscope. Each experiment was performed in triplicate (n = 3 in each experiment)

### Seahorse XF24 metabolic flux analysis

Seahorse XF Mito Stress Test Kit (Cat # 103015–100, Agilent, Santa Clara, USA) was used to measure oxygen consumption rates (OCR, pmol/min) and extracellular acidification rates (ECAR, mpH/min). One day prior to the experiment, 6 X 10^4^ cells/ well were seeded in a XF24 cell culture plate in complete DMEM/F12 media. For D425, the wells were first coated with polylysine. On the day of the experiment, cells were washed and incubated in XF assay medium supplemented with 17.5 mM glucose, 2.5 mM glutamine, and 0.5 mM sodium pyruvate for 1 hour at 37°C in 0% CO2. Mitochondrial inhibitors: oligomycin (1μM), FCCP (0.5 μM) and rotenone/actinomycin A (1μM) were added based on the manufacturer’s recommendation. After the experiment, all the cells were recovered, and OCR/ECAR measurements were normalized to protein content per well using the BCA protein assay kit (Thermo Fisher Scientific).

### Intracranial xenograft model

Xenograft tumor cell suspension was carried out in 6–8-week-old NSG mice (NOD-SCID gamma mouse). Mice were obtained from the laboratory breeding colony, established by breeding pairs purchased from Jackson Laboratories. All procedures were reviewed and approved by the UCI Institutional Animal Care Use Committee. Sample sizes were chosen to minimize the number of animals required to achieve significant results.

One week prior to the cell injection, animals were surgically implanted with a catheter into the jugular vein, as previously described [25]. Briefly, catheters were constructed with the guide cannula bent at a curved right angle and fitted with 6 cm silastic tubing. For the intravenous surgery, the catheter port was subcutaneously implanted in the animal’s back, and tubing was guided under the skin at the shoulder/neck to the right jugular vein. Thereafter, 1 cm of the tubing was inserted into the vein and secured with surgical silk suture [25]. On the day of stereotactic implantation, mice were anesthetized by isoflurane/oxygen vapor mixture. An incision of ~1.5 cm was made along the mediolateral line near the back of the skull using a scalpel (Feather Safety Razor Co.). Once the area was clean, a burr hole was drilled using a handheld microdrill. D425 cell suspension (2 μl; 1.75 x 10^5^ cells) was injected into the cerebellum of 19 mice using a 30G Hamilton Syringe (7803–07, Hamilton Company) with stereotaxic guidance (AP: - 2mm; DV: -2mm; ML: 1mm, relative to lambda). The needle was left in place for an additional minute to limit reflux along the injection site. Following closure of the incision, mice were removed from isoflurane and injected subcutaneously with 10 mg/kg carprofen and 10mg/kg enrofloxacin immediately after the surgery and 5 days later. Seven days after the surgery, animals received 50 mg/kg BT9 intravenously three times per week until they were euthanized. Captisol was used as control. After orthotopic transplantation, mice were observed daily for moribund signs which included weight loss, haunching, impaired motility and breathing, which prompted euthanasia procedures. Mice with weight loss equal to or greater than 20% were automatically euthanized in accordance with UCI IACUC regulations. The mice eligible for euthanasia were given a 150 mg/kg dose of Euthasol by I.P injection and placed in a cage to observe respiratory rate until breathing stops. Mouse stimulation was assessed by toe pinch or other method before proceeding to cardiac perfusion or secondary method to ensure death.

### Statistical analysis

Statistical analysis was performed using GraphPad Prism 9 software. Group comparisons were performed using Student’s t-test or ANOVA based on whether two or more groups were compared, respectively. The Mann-Whitney test was used for group comparisons. P < 0.05 was considered significant. Survival analysis was performed using Kaplan-Meier.

## Results

### MAGMAS inhibition reduces cell proliferation and induces cell death in both SHH-driven and G3 medulloblastoma cell lines

**Fig 1:**
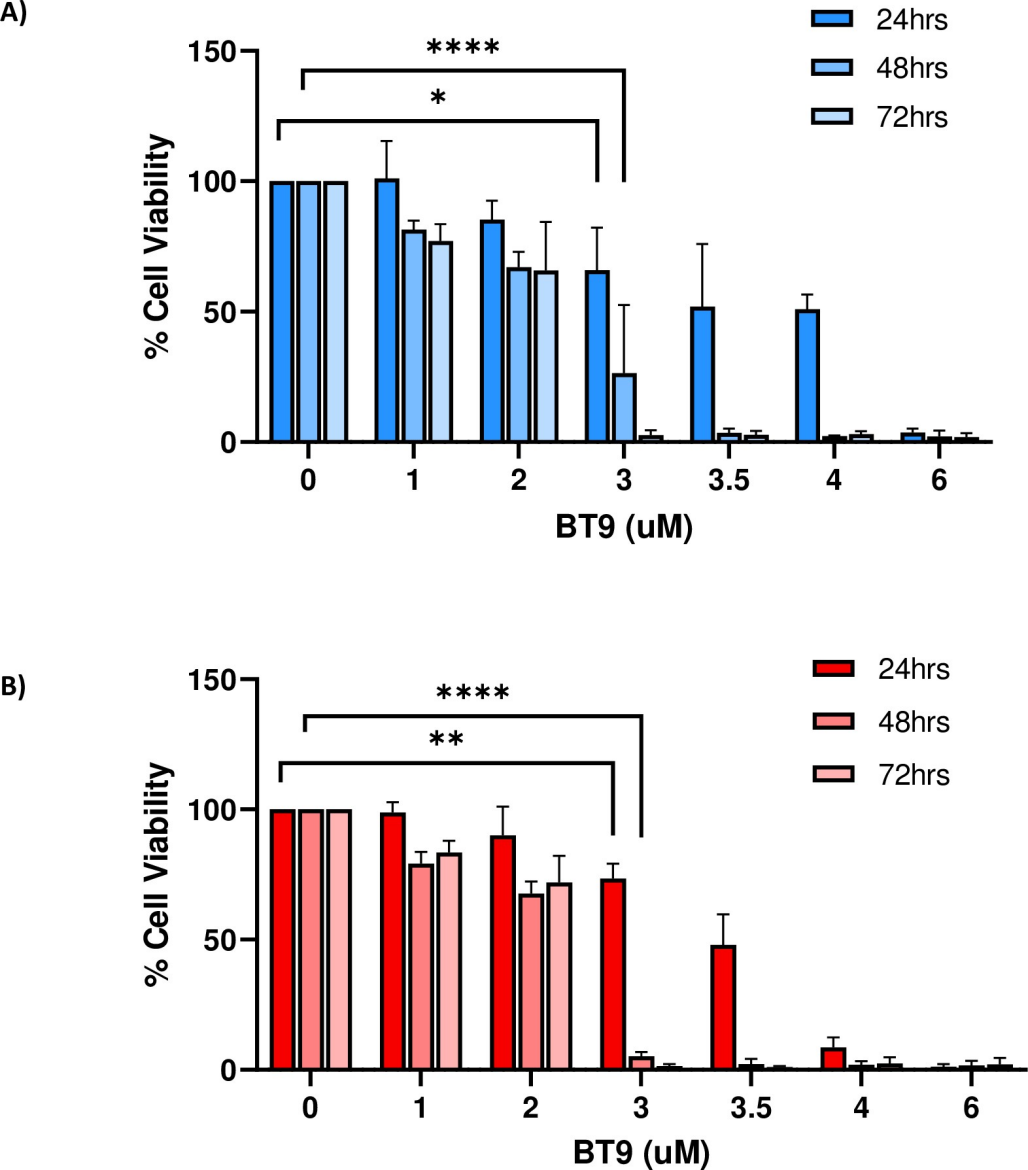
BT9 exhibits a dose- and time-dependent cytotoxic effect on medulloblastoma. DAOY **(A)** and D425 **(B)** were incubated for 24, 48, and 72 hours with increasing concentrations of BT9. Cell viability was measured by MTT assay. The relative numbers of proliferating cells in each condition were compared to control and are presented as the mean ± SEM. *p < 0.05, **p < 0.01, ****p < 0.0001.

**Fig 2:**
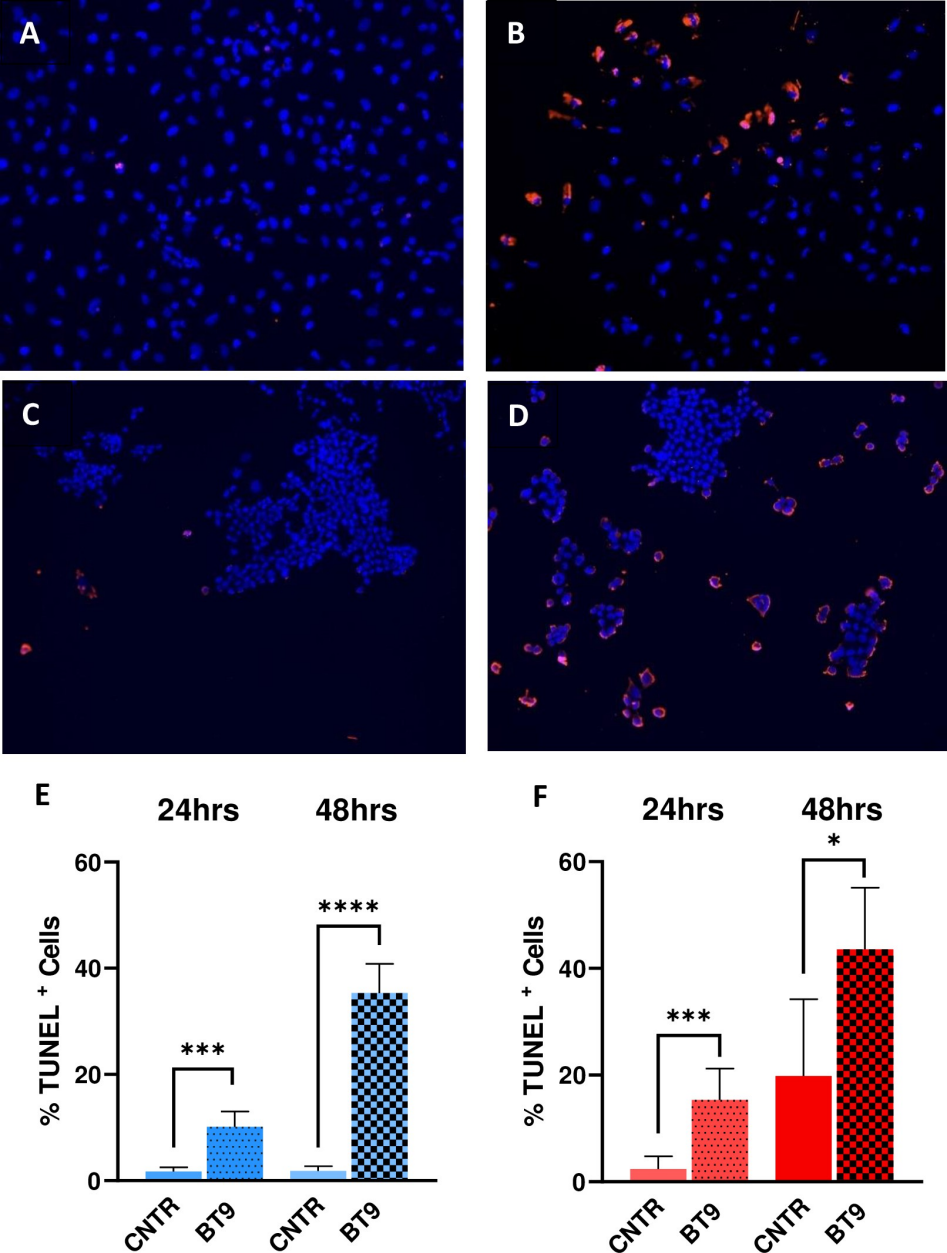
MAGMAS inhibition induces medulloblastoma cell death. Representative images show DAOY (A and B) and D425 (C and D) cells treated with vehicle control (A and C) or BT9 (B and D). Quantification of TUNEL-positive DAOY (E) or D425 (F) cells after 24 and 48 hours of BT9 treatment compared to control (*p < 0.05, ***p < 0.001, ****p < 0.0001, n = 9). 10x magnification.

To test the effects of BT9 on cell proliferation, we performed MTT assay on established medulloblastoma DAOY and D425 cell lines. The cells were treated with increasing concentrations of BT9 at three different time points, 24, 48, and 72 hours. As shown in Fig 1A, BT9 treatment significantly inhibited DAOY cell growth in a dose- and time-dependent manner (24 hours IC50: 3.6 μM, 48 hours IC50: 2.3 μM, 72 hours IC50: 2.1 μM). Similar sensitivities were observed on D425 cells (Fig 1B, 24 hours IC50: 3.4 μM, 48 hours IC50: 2.2 μM, 72 hours IC50: 2.1 μM). To determine whether decreased cell proliferation as due to induction of cell death, we performed the TUNNEL assay. Consistently, BT9 treatment led to a significant increase in the proportion of TUNNEL-positive cells in both DAOY (Fig 2A, 2B and 2E; control 1.7% (171/11348 cells) vs BT9 10.1% (759/7686 cells), p = 0.0004, 24 hours; 1.8% (165/9224 cells) vs. 35.3% (1926/5603 cells), p <0.0001, 48 hours) and D425 (Fig 2C, 2D and 2F; control 1.4% (87/6250 cells) vs. BT9 (1306/ 8542 cells) 15.3%, p = 0.0001, 24 hours; 19.8% (1503/7591 cells) vs 43.6% (5338/12532 cells) p = 0.02, 48 hours) cells. In summary, we found that BT9 prevents medulloblastoma cell proliferation by inducing cell death

### BT9 decreases medulloblastoma cell migration and invasion

**Fig 3:**
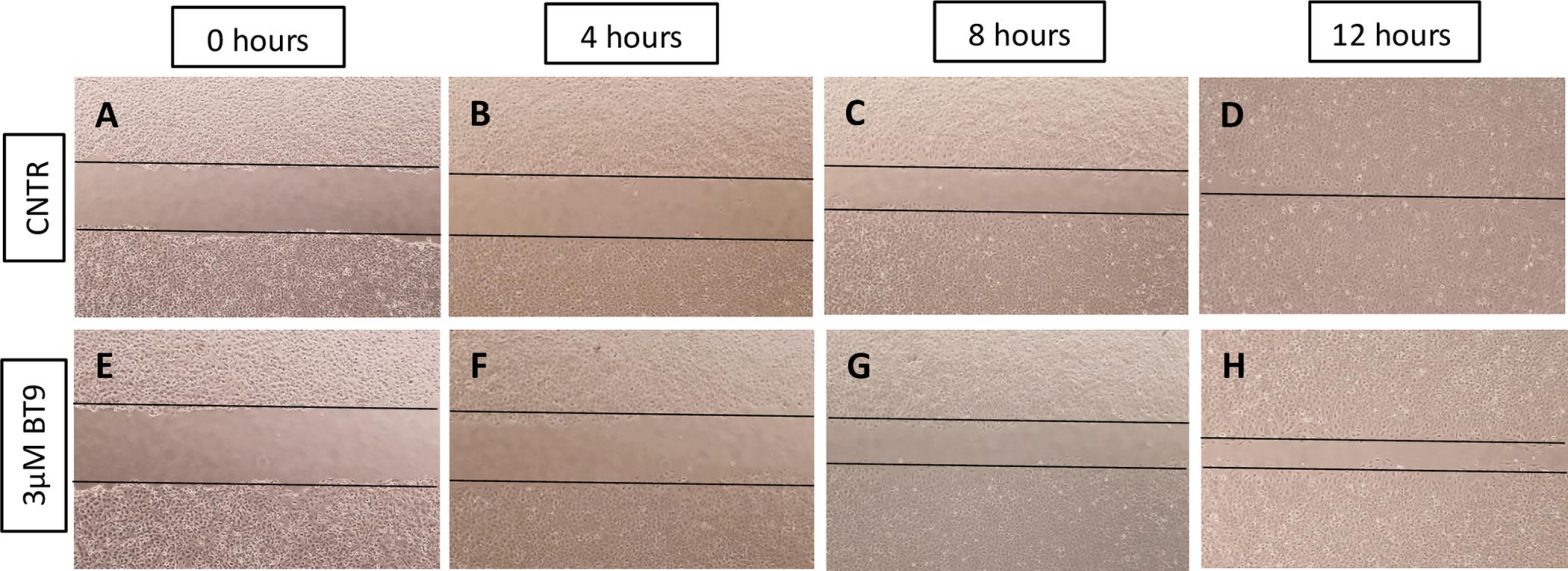
MAGMAS inhibition reduces DAOY cells migration. The scratch wound assay was used to compare the effect of control (A-D) or 3 μM BT9 (E-H) on DAOY cell migration. Representative photographs were taken at T0, T4, T8, and T12 after the scratch. 4X magnification.

We next examined the effect of BT9 on DAOY cell migration using the scratch wound assay. Representative images (Fig 3A–3H) depict control (Fig 3A–3D) versus 3 μM-treated BT9 cells (Fig 3E–3H) at 4, 8, and 12 hours after performing the scratch. Our results showed that BT9 delayed migration of DAOY cells into the center of the wound. Due to the non-adherent nature of D425, we were not able to perform the scratch assay on this cell line.

**Fig 4:**
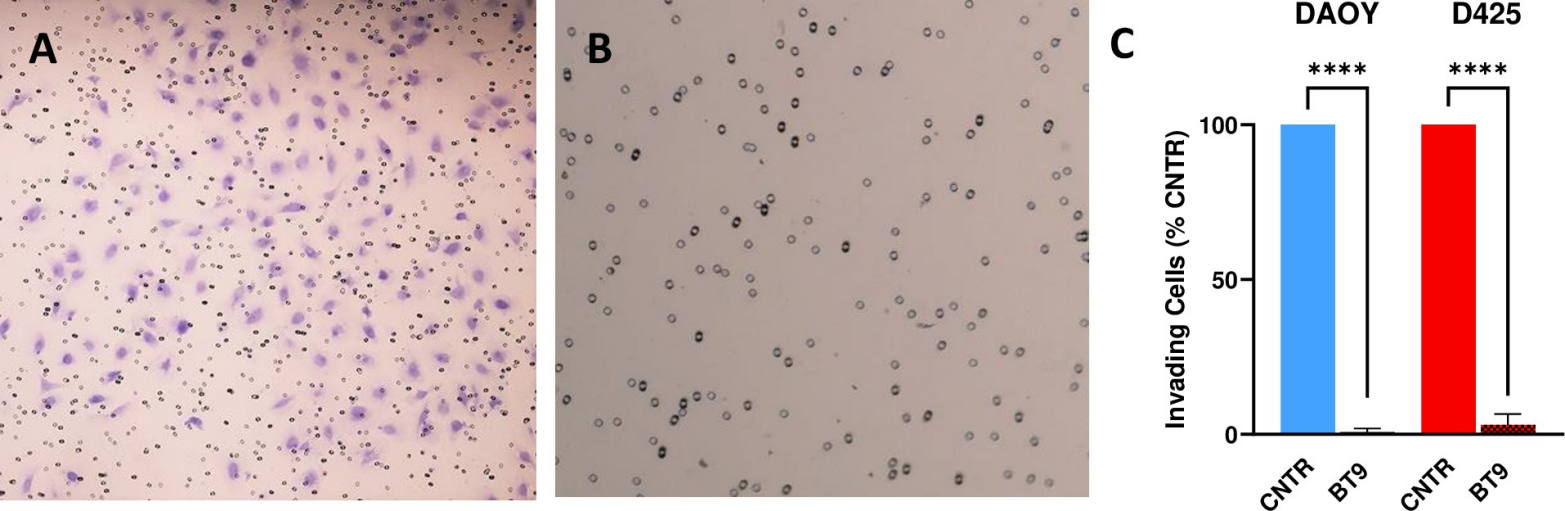
BT9 reduces DAOY and D425 medulloblastoma cell invasion. (A and B) Representative images show the invading DAOY cells (purple cells) in vehicle (A) or 3μM BT9-treated (B) cells. (C) Compared to control, BT9 treatment significantly decreased the number of invading DAOY and D425 cells (****p < 0.0001, BT9 vs vehicle control, n = 6). 4x magnification.

To better understand the effect of BT9 in inhibiting cell movement, we performed as invasion assay with Matrigel-coated inserts, which mimic the natural barrier that cells must overcome to invade into other tissues. Consistent with our migration data, BT9 treatment resulted in a significant reduction in the invasive capacity of both DAOY and D425 cells compared to control after 22 hours (Fig 4A–4C, DAOY, BT9/control: 27/5120 cells; D425, BT9/control: 11/2115 cells). Together, we found that BT9 can effectively inhibit medulloblastoma cell migration and invasion.

### MAGMAS inhibition in medulloblastoma cell lines alters mitochondrial respiration

**Fig 5:**
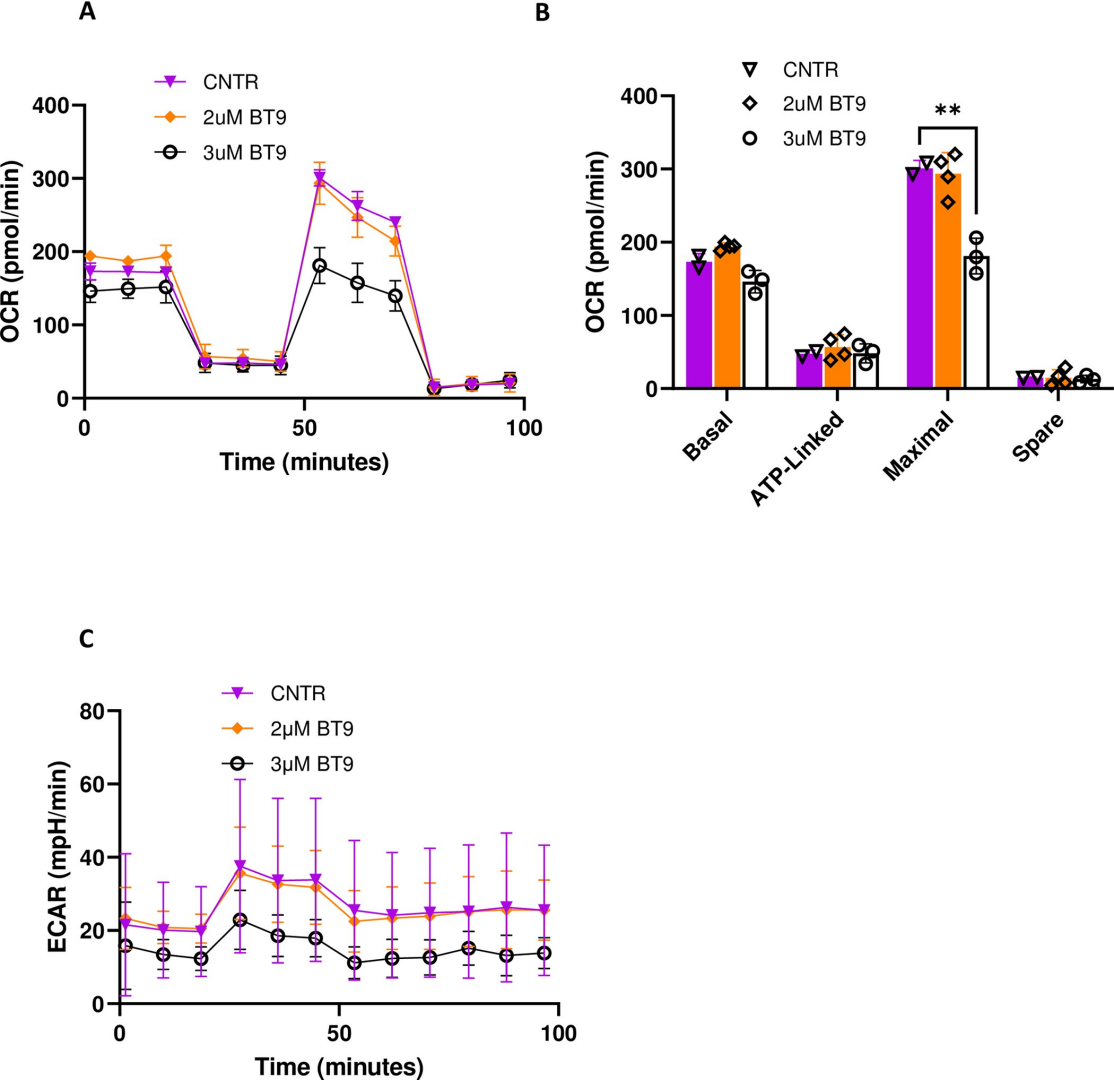
The effect of MAGMAS inhibition on mitochondrial respiration and acidification rate in D425 cells. (A) Oxygen consumption rate (OCR) profile of D425 treated with 2 and 3 μM BT9 for 24 hours. The result showed a decline in oxygen consumption of D425 cells treated with 3μM BT9. (B) Bar chart showing the results of mitochondrial respiration changes in BT9-treated cells, which were analyzed with basal respiration, ATP production, maximal respiration, and spare respiratory capacity. (C) Extracellular acidification rate (ECAR) profile of D425 treated with 2 and 3μM BT9 for 24 hours (**p < 0.01, 3μM BT9 vs control (CNTR).

MAGMAS promotes cellular tolerance toward oxidative stress by increasing electron transport chain (ETC) function and thus reducing ROS production [14]. To measure the effect of MAGMAS inhibition on mitochondrial respiration, we assessed cellular oxygen consumption rate (OCR) and Extracellular Acidification Rate (ECAR) using the Seahorse Biosciences Extracellular Flux Analyzer. 3 μM BT9 treatment for 24 hours led to a decline in OCR and ECAR in D425 (Fig 5A and 5C). Upon examining the key parameters of mitochondrial functions, encompassing basal, ATP-linked, maximal, and spare capacities, a significant reduction was observed in maximal activity of mitochondria (p = 0.003) (Fig 5B). We observed minimal effect on DAOY cells (S3A and S3B Fig) cells.

### BT9 does not extend survival in a D425 medulloblastoma animal model

**Fig 6:**
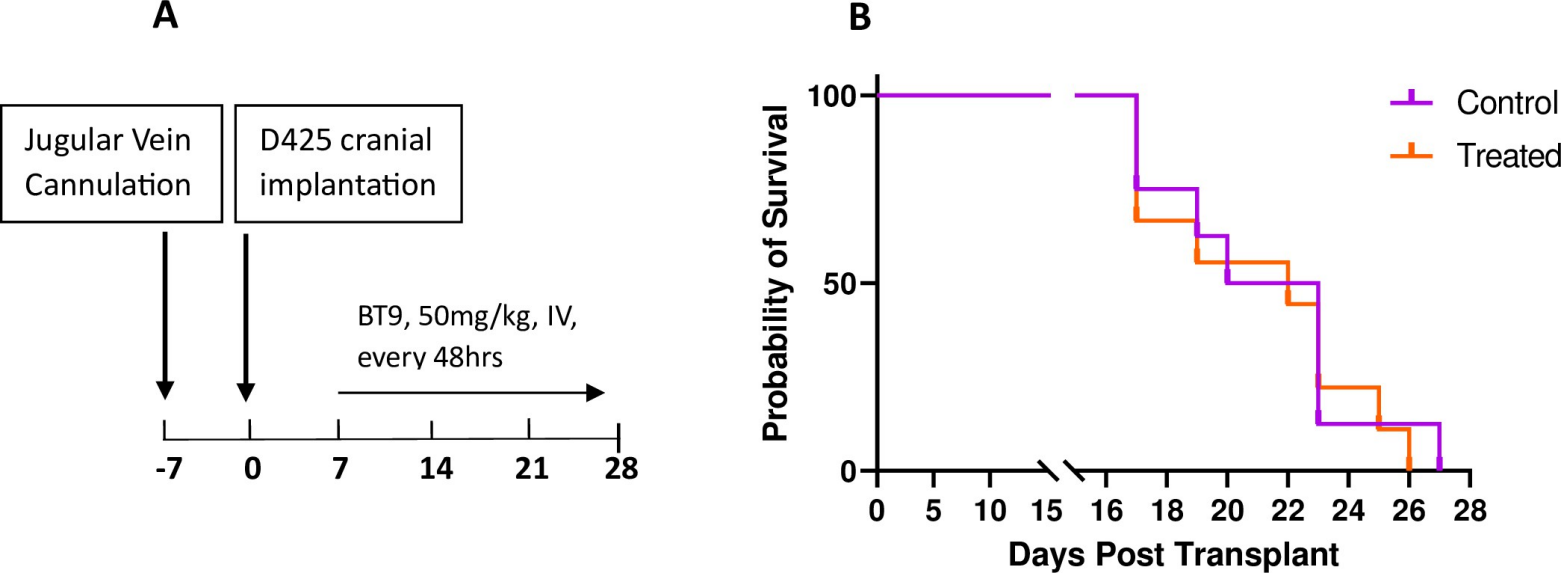
BT9 did not improve survival in the orthotopic xenograft model of D425 medulloblastoma. (A) Experimental design. Immunodeficient (SCID) mice were surgically cannulated in the jugular vein. One week after, they were intracranially implanted with D425 cells. Seven days later, each animal started to receive intravenous injections of 50 mg/kg BT9 or vehicle control (Captisol) every 48 hours until they were euthanized. (B) Kaplan-Meier survival analysis. BT9 treatment did not increase survival in experimental group compared to vehicle-treated controls (27 days; n = 8 control vs. n = 9 treated group).

Finally, we investigated the effect of BT9 treatment as monotherapy in a D425 orthotopic xenograft mouse model. 7 days after cell implementation, 50 mg/kg BT9 was given intravenously through the jugular vein, three times per week, until the animals showed signs of cancer development and were euthanized (Fig 6A). We did not observe any significant extension in survival of the BT9-treated animals (Fig 6B).

## Discussion

Mitochondria are essential organelles serving not only as energy factories but also as key players in cell proliferation and death. Alterations in mitochondrial metabolic and signaling pathways have been associated with both tumorigenesis and cancer progression [26]. In recent years, mitochondria have emerged as a therapeutic target in many types of cancers, such as breast, lung, colon, and pancreas [26–28]. These agents target various mitochondrial functions such as metabolism, apoptotic pathways, and ROS homeostasis, and their potential efficacy is actively explored [28].

BT9 is a novel small molecule that binds to MAGMAS and potentially blocks protein trafficking across the inner membrane, and it has shown inhibitory effects on proliferation and migration in glioblastoma and prostate cancer cell lines [19,29]. In this study, we investigated the efficacy of BT9 in pediatric medulloblastoma models.

We demonstrated that BT9 reduced cell proliferation in SHH-activated/tp53 mutant and G3 medulloblastoma cells. In our study, both DAOY and D425 cells showed the same sensitivity to MAGMAS inhibition (3.64 vs. 3.38 μM at 24 hours, 2.28 vs. 2.17 μM at 48 hours, and 2.14 vs 2.16 μM at 72 hours, respectively). These effects are consistent with our results in glioblastoma, where IC50 was found to be approximately 2.5 μM after 72 hours of BT9 treatment [19]. We also found an increased frequency of cell death in BT9-treated cells; this effect was directly correlated with caspase level. It is also possible that BT9 induces cell death in medulloblastoma via other pathways. Yang et al. has reported both caspase-dependent and -independent pathways as the modes of BT9-induced cell death in prostate cancer cells [29].

The disruption of mitochondrial function plays a crucial role in initiating apoptosis. Furthermore, apoptotic cells exhibit a distinct metabolic activity pattern that differentiates them from healthy cells. At early stages of apoptosis, cells generally maintain the ATP level, which is essential for the execution of the apoptotic program [30]. However, as the apoptosis progresses both OCR and ECAR decrease. Measuring mitochondrial respiration in D425 cells, BT9 failed to impact mitochondrial parameters besides diminishing maximal, which represents the upper limit of mitochondrial respiratory capacity. Certain drugs such as uncoupling proteins has been shown to reduce the maximal respiratory capacity of mitochondria without affecting their basal respiration or ATP production under normal conditions [31]. In these conditions, the mitochondria would still function normally for routine cellular activities but would be unable to respond to increased energy demands or stressors by increasing their respiratory capacity. Our findings show that BT-treated cells have less respiratory reserve available for meeting increased energy demands or coping with stress, which could lead to apoptosis. The absence of an effect at 2μM may result from a compensatory rise in metabolic activity aimed at producing sufficient ATP for apoptosis induction. Furthermore, we detected a reduction in ECAR, which reflects proton (H⁺) production and secretion into the extracellular environment and is mainly driven by glycolysis [32]. Although a decrease in OCR generally correlates with an increase in ECAR, the relationship is not absolute. For example, treatment with metabolic inhibitors such as oligomycin that target mitochondrial respiration can simultaneously decrease OCR and ECAR [33,34]. Prolonged exposure to toxins that overcome glycolysis’s ability to meets the cell’s energy demands can also lead to metabolic exhaustion and subsequent decline in ECAR [34]. This could explain the simultaneous reduction in both OCR and ECAR in D425 after BT9 treatment. On the other hand, OCR and ECAR declines might also reflect the increased number of apoptotic cells. Finally, we observed a lower OCR baseline in D425 (173 pmol/min) compared to DAOY (659 pmol/min) cells (Figs 5A and S3A). These findings are consistent with recent data where Li et al. demonstrated that mitochondrial-encoded oxidative phosphorylation complexes are the most downregulated genes in G3 medulloblastoma [35]. We also found that BT9 reduces the migration and invasion of medulloblastoma cells, potentially also attributable to its impact on the mitochondrial metabolism.

Despite promising *in vitro* results, we did not find any significant increase in survival in the D425 medulloblastoma *in vivo* model after BT9 treatment. This result does not completely negate the efficacy of BT9 in the *in vivo* system. It simply shows that under the protocol used in this study, BT9 is not effective, and further optimization is required. We suggest using higher (>50 mg/kg) and/or more frequent (ex. daily) doses of the BT9. We started BT9 treatment 7 days after cell implantation. One might try to administer the drug at earlier time points. It is possible that the tumor had already progressed to a late-stage cancer by the first date of our treatment. We also suggest validating BT9 efficacy in patient-derived xenograft models. Finally, given that BT9 will likely be part of a broader treatment regimen [36], we recommend the investigation of BT9 in combination with other chemotherapeutic agents. In fact, we found that BT9 shows additive effects when combined with temozolomide in glioblastoma cells (unpublished data). In line with this, Ahmed et al have reported MAGMAS inhibition as an effective treatment strategy for patients with chemotherapy-resistant ovarian cancer [18].

In conclusion, our results indicate that inhibiting MAGMAS hinders cell proliferation, migration, and invasion by disrupting mitochondrial function. Further studies are needed to confirm its potential efficacy, either as a standalone or as a combination therapy.

## Supporting information

S1 Fig PAM16 correlation with N-MYC. Data curated in GlioVis using Northcott 2012 study data sets (http://gliovis.bioinfo.cnio.es/). The light red line represents the confidence intervals (>95%) [37].
(TIF)

S2 Fig Expression of MAGMAS in medulloblastoma. Microarray data show overexpression of PAM16 in medulloblastoma subtypes. Data curated in GlioVis using Northcott 2012 study data sets (http://gliovis.bioinfo.cnio.es/) [37].
(TIF)

S3 Fig MAGMAS inhibition on mitochondrial activity in medulloblastoma cells. (A) OCR and (B) ECAR profile of DAOY treated with 2 and 3 μM BT9 for 24 hours. The result does not show a significant effect on the mitochondrial activity.
(TIF)
