# Supplementary figures and images for: Preclinical assessment of MAGMAS inhibitor as a potential therapy for pediatric medulloblastoma
Source: PLoS One. 2024 Oct 22;19(10):e0300411. doi: 10.1371/journal.pone.0300411 (PMC11495579; doi:10.1371/journal.pone.0300411)

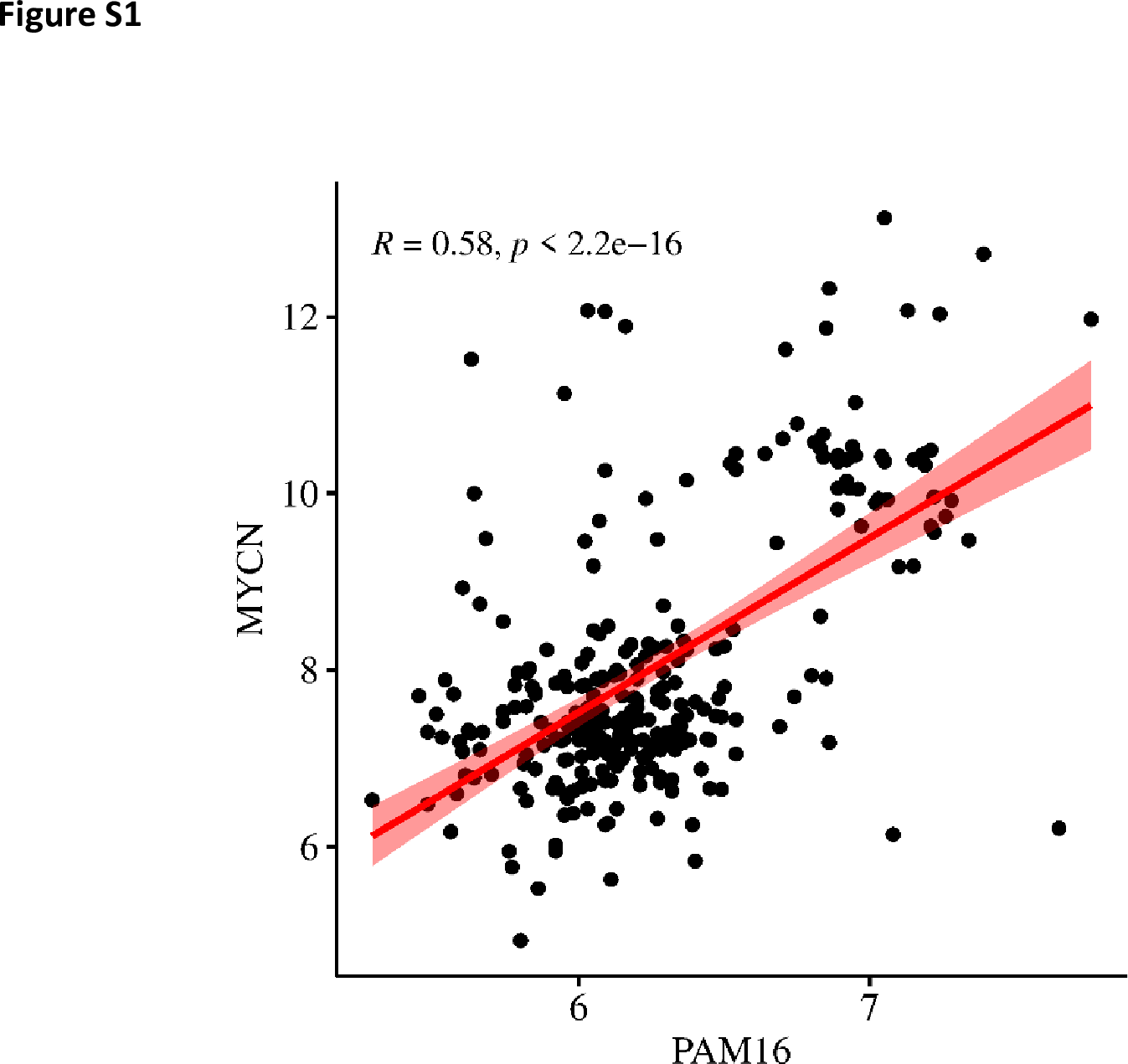

Supplement: S1 Fig — Data curated in GlioVis using Northcott 2012 study data sets (http://gliovis.bioinfo.cnio.es/). The light red line represents the confidence intervals (>95%) [37]. (TIF) [file pone.0300411.s001.tif]

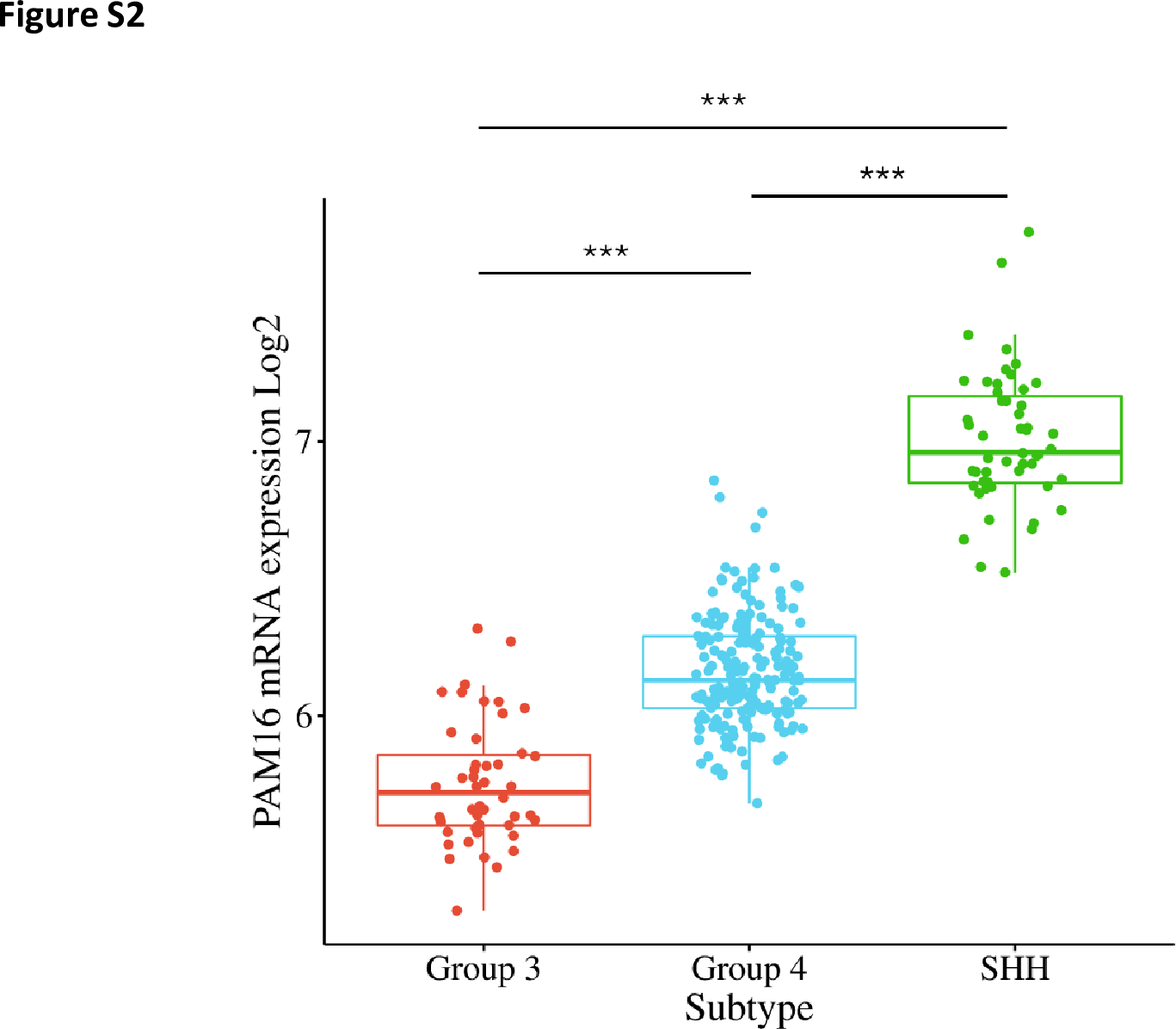

Supplement: S2 Fig — Microarray data show overexpression of PAM16 in medulloblastoma subtypes. Data curated in GlioVis using Northcott 2012 study data sets (http://gliovis.bioinfo.cnio.es/) [37]. (TIF) [file pone.0300411.s002.tif]

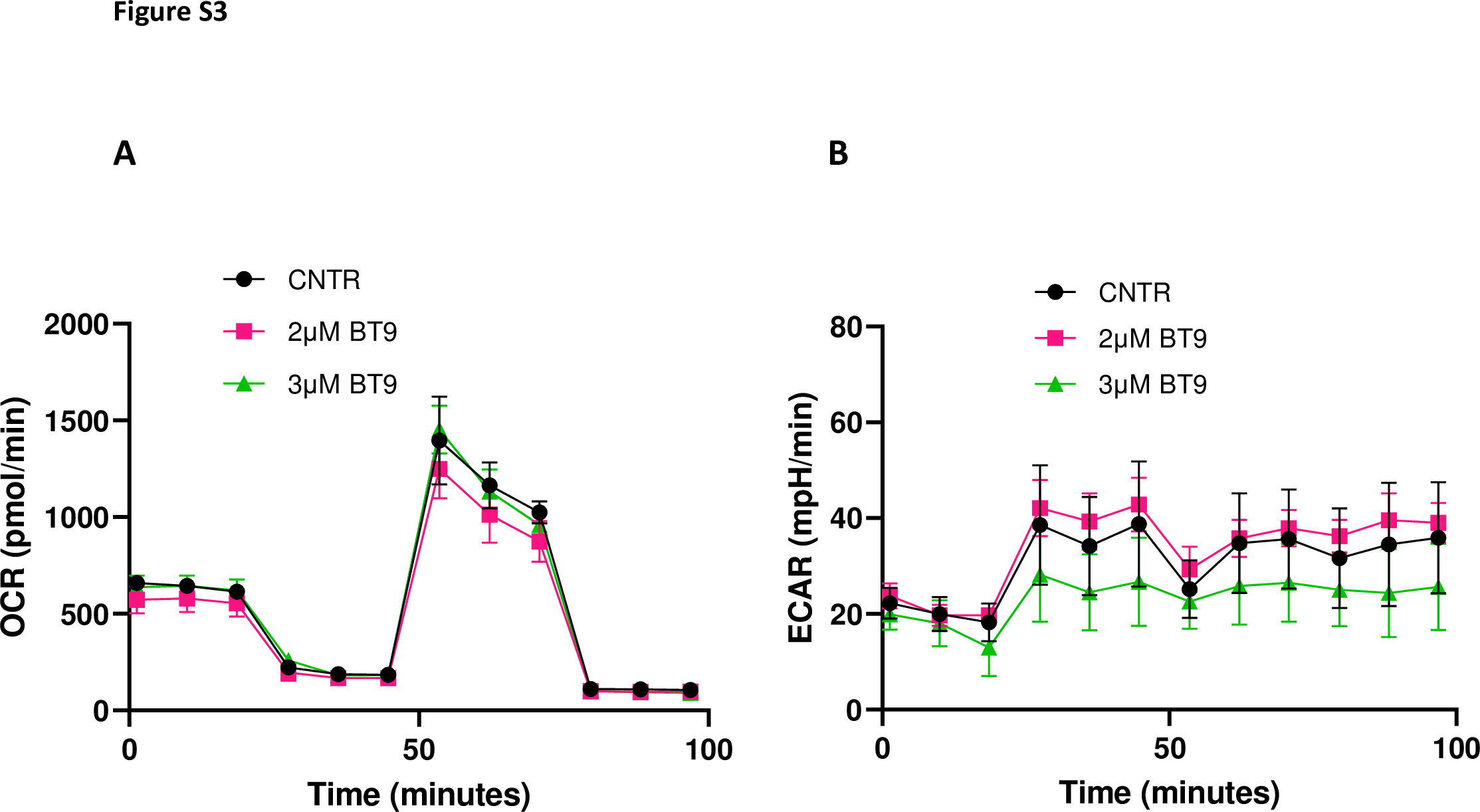

Supplement: S3 Fig — (A) OCR and (B) ECAR profile of DAOY treated with 2 and 3 μM BT9 for 24 hours. The result does not show a significant effect on the mitochondrial activity. (TIF) [file pone.0300411.s003.tif]
